# Restoring the Double Disconnect: Towards a Conceptual Reinstatement of Opiate Addiction as a High Risk Behaviour

**DOI:** 10.5812/ijhrba.6936

**Published:** 2012-07-25

**Authors:** Albert Stuart Reece

**Affiliations:** 1 School of Psychiatry and Clinical Neurosciences, University of Western Australia, Crawley, Australia

**Keywords:** Ageing, Opiate Dependence, Heroin, Methadone, Pathophysiology, Opiate Toxicology

For many years it has been well known, and well documented that the majority of the world’s heroin originates in Afghanistan (1). For nations immediately adjacent to this major source, and for nation on the overland distribution routes to Europe, this poses an almost insuperable public safety and law and order challenge related to the virtual impossibility of close control of land borders. The enormous public health challenges posed by an uncontrolled opiate influx are further amplified by the weak medical consensus relating to the medical consequences of long term clinical opiate dependence (COD). This poses an immediate and severe apparent paradox: whilst the effects of long term opiate dependence on the majority of its users are all too obvious to the general public, many sources of expert medical opinion deny that there are in fact any significant health impacts associated with long term opiate dependence (2).

This contradictory scenario, which is the common situation in most developed nations, sets up a double disconnection: firstly between the general public and the medical profession which guard and advise their communities; and secondly within the medical profession itself where the all too obvious physical and psychiatric ravages of long term drug addiction have been routinely denied for decades. It is the central thesis of this editorial that the resolution of this double dysjunction is to be found by an improved understanding of the long term toxic pathophysiology of opiate dependence by the medical profession based on a greater appreciation of old and new insights from researchers in the basic sciences informing clinical practice and public health policy. This renewed, revitalized and refreshed clinical appreciation can then more rightly inform public health approaches and treatment programs, to properly advise and warn the general public.

 In considering the epidemiological impacts of chronic opiate abuse it is instructive to consider first some major overarching multi-systems contributions. As long ago as 1877 Claude Bernard wrote a whole chapter in his book on diabetes based on the common understanding at that time that opiates would acutely induce a hyperglycaemic diabetes-like state (3). This observation, now long forgotten, has proven to be profoundly prescient given our current understanding of diabetes as frequently being a degenerative disease of ageing, a disorder of dysfunctional immunity, and a disorder of impaired stem cell regeneration. More recently, Australian workers reviewing a 21 year history of a methadone maintenance program, showed that long term dependency was associated with elevated mortality rates from neuropsychiatric, cardiovascular, gastro-intestinal, respiratory and cancerous diseases (4). This group has also shown rates of severe coronary disease of 17% in COD more than 44 years of age, and shown that the impacts of methadone on single organ systems (OR = 4.13) and multiple organ systems (OR = 3.11) is more severe than heroin (5). Workers in California found rates of heart and liver disease, stroke, cancer and suicide elevated 3-30 times (6). Rosen, also in the US, found that opiate dependent patients of 50 years or more had worse functioning than an older control group of the general community in all eight areas of physical and mental function assessed (7), to the point where she called for COD older than 50 years to have a geriatrician appointed to their care due to the accelerated appearance of the disorders of old age. 47% or her group required psychotropic medication, and 18% of African-Americans self-reported diabetes.

Negative health effects of opiates have been reported with atherosclerosis (8), osteoporosis (9), immune dysfunction (10), hair greying (11), stem cell deficiencies (12) and cancers (6, 13). Atherosclerosis and osteoporosis are well defined age related disorders. 83% of a Boston study had reduced bone mass (9). COD demonstrate the polyclonal gammopathy seen in high risk nonagenarians facing a 2-year mortality of over 60% (14). Hair greying is the sine qua non of human ageing, and its three fold high rate in COD strongly implies accelerated ageing processes in these patients (11). Lower stem cell counts in opiate dependence also suggest accelerated age related change (12). The cardiovascular surgical group from Tehran have shown that COD is associated with an odds ratio of coronary disease requiring revascularization of 1.8, demonstrated a dose-response relationship, preceded that developing in non-COD by four years, and was independent of tobacco consumption (8, 15). Series of increased rates of lung, liver and anogenital (including cervical) (4, 16), larynx, esophagus, and bladder have been published. Careful work from an Iranian group showed that rates of laryngeal cancer elevated 25-fold were independent of the effects of tobacco (13).

Clearly such a widespread pattern of generalized organ dysfunction strongly suggests that common underlying mechanisms of cellular cytotoxicity might be at play to account for these observed phenomena. The first such mechanism has been studied since the 1970’s and has now been shown to be related to the specific binding of opiates to the perinuclear receptor called the opiate growth factor receptor (OGFr) which has been cloned and sequenced at both the protein and gene levels. Upon ligation the OGFr migrates to the nucleus and stimulates the major blocks to cell cycle renewal by P16 inhibitor of cyclin dependent kinase 4A (P16INK4A) and cyclin dependent kinase inhibitor 1 (P21 WAF1/CIP1) (17) at the G1/S checkpoint. This effect is exacerbated by an effect of opiates to precipitate or predispose towards apoptosis or cell death. Dividing cells, including stem cells, are known to be particularly susceptible to immune activity (18).

The other area of great research interest in modern times is the direct neuroimmune effects of opiates. It has been shown that the morphine an opiate nucleus binds directly to the endotoxin grove of myeloid differentiation factor 2 (MD2) which is the extracellular binding partner of the toll-like receptor (TLR4). In doing so it directly triggers the powerful TLR4 - NF-κB (nuclear factor kappa –B) immune signalling pathway and synthesis and release of the pro-inflammatory pre-proteins interleukin (IL) -1 and -18. Inflammatory and stressful activity in the extracellular and intracellular milieu trigger inflammasome formation by multiple pathways and so the active native forms of the protein are cleaved from their pre-cursors and the powerful inflammatory cascades downstream of these sequences are unleashed. The point is that at the juncture of inflammasome function, inflammatory, stress, and cell death and cell damage signals are integrated and powerfully amplified both in the brain in all other organ systems (19). Clinical studies showing elevated levels of TNFα (tumour necrosis factor-α) and IL-1β in COD strongly endorse this schema (10) as does a wealth of recent basic sciences research.

In addition to binding at classical μ, γ, κ and orphanin/nociception receptors, opiates have also been shown to bind to the mitogen activated protein kinase (MAPK), transforming growth factor-beta (TGFβ), and phosphoinositol-3 kinase (PI3K) pathways, all of which are critically involved in cell renewal – cell death decisions and classical oncogenic pathways. Taken together this under-recognized epidemiological and molecular body of evidence strongly suggests that the true toxicity of long term opiate addiction has been seriously underestimated by mainstream medical practice. Moreover if COD were thoroughly and rigorously investigated and understood at the molecular level our appreciation of the basic biology of degenerative disorders including atherosclerosis, neuropsychiatric disease, cancer and ageing would almost certainly be improved. The advent of subcutaneous naltrexone implants, which reverse the pathophysiology of COD, and can act for up to two years, creates a major therapeutic alternative to indefinite opiate maintenance which in many places has become a treatment of last resort, and is closely aligned with a philosophy of therapeutic despair and nihilism. In other words advances in general biology will inform our understanding of the treatment of opiate dependency, and advances in our understanding of the pathophysiology of opiate dependence will inform our biological perceptions, and indicate potential therapeutic targets for many age related disorders such as those alluded to above. 

The key to unlocking this puzzle will be dedicated research coupled with open dissemination of information first throughout the medical profession and then to the wider community beyond. The treatment of both COD and age related disease in the general community can be expected to benefit.
